# The Twentieth Century

**Published:** 1901-01

**Authors:** 


### Editorial.

### THE TWENTIETH CENTURY.

1901. The sound seems significant of youth, infancy, the first year of life. The age of humanity, when the mother thinks of her wailing infant and the father wonders whether the horoscope of the life will be for the happiness of the child. The dentist thinks of the evolution of teeth and dentition.

The first year of a century is to the sentimental mind the reincarnation, as it were, the unfolding of a new life, but the practical man regards it as simply the recrudescence of all that has gone before.

We celebrate the years of our birth and make merry at each returning Christmas, and become saddened as each annual day brings to memory the departure of some loved one to join the immortals. Thus humanity is ever breathing a new inspiration and taking up the old burdens, waiting for the end it sees not.

The century, the nineteenth of the Christian era, closed with December, and a portion of the world began anew to dream dreams of the possible future that lies locked up in the unknown years that will mark another century upon the calendar of a world's development.

The prophet is not born that can tell what these years may bring forth for dentistry. The thought has been, heretofore, expressed on these pages that it might in the near future be lost as a distinct profession. It is possible that this may happen, but whether dentistry dies by name or not, stomatology will exist as long as teeth are necessary for mastication. The dentistry of the future may be known by another name, but those who follow it will still be laboring to save the teeth of the human race, and, failing in that, will still be inserting substitutes, crowns, bridges, and artificial dentures.

It requires no prophet to foretell that the mind of the future will become more and more intelligent regarding teeth, causes of decay, and the importance of proper sanitary measures to prevent destruction. It needs no keen foresight to know that a portion of the general training of the future will be in the direction of prophylactic measures to insure health. The man of the last years of the twentieth century should be able to so care for himself that sickness should be something of a discredit, conveying the impression that there had been a lack of care, a want of cleanliness, and a disregard of the laws of life fairly well understood before the curtain is rung down on two thousand years.

The world hopes much for the incoming time. The past is secure in its triumphs. The present generation may rejoice in that it took part in all that made the nineteenth century glorious, but as the days drew to a close the thoughtful, altruistic mind could not but feel that this century had failed to make for a much higher civilization than that of the fifteenth. The wars and rumors of wars that have disgraced the declining months of this noble epoch are the lingering evidences of the brutality in human nature that seems to place far away the time when spears may be formed into pruning-hooks and swords into ploughshares.

While we thus face the first twelve months of another century, and may look forward hopefully for the further development of our loved profession, let us not forget that the skill obtained has been the result of a self-sacrificing effort on the part of many men. That if the eighteenth and nineteenth centuries made dentistry, to the twentieth belongs the responsibility of so completing the work that whether it lives under that name or not, it will still be known as a living fact in the life of all civilizations.

Let us then gird ourselves anew and resolve that whether our days be many in the land, or otherwise, we will never be drones in the busy hives of a world's activities. The responsibility of the future of dentistry belongs in part to every reader of this journal. It cannot be placed on some one else. The accountability comes directly home to you. There is much work to be accomplished. Your journal needs your best thought and your most earnest effort for its success. Your society needs your constant help. Your State organizations will suffer without your aid, and the National organization looks to you to give it your undivided allegiance. More than all this, the century demands of you that you will so conduct your professional life that it will be honorable before the world, and in that proportion will dignify your calling. The twentieth century demands this at your hands, and in so far as you fail to meet its requirements will you do your part to destroy the results that were given to dentistry by those who worked and toiled and self-centred their lives that their profession might be respected among men.

